# Hemodialysis Record Sharing: Solution for Work Burden Reduction and Disaster Preparedness

**DOI:** 10.2196/32925

**Published:** 2022-07-22

**Authors:** Keisuke Ido, Mariko Miyazaki, Masaharu Nakayama

**Affiliations:** 1 Medical Information Center, Tohoku University Hospital Sendai Japan; 2 Division of Nephrology, Endocrinology and Vascular Medicine Tohoku University Hospital Sendai Japan; 3 Department of Medical Informatics Tohoku University Graduate School of Medicine Sendai Japan

**Keywords:** hemodialysis, electronic health record, EHR, information sharing, information exchange, data sharing, document sharing, health information exchange, disaster, work burden, clinical information, clinical data, clinical report, medical report, information network, medical informatics, renal failure, kidney, renal, clinical record, medical record, backup, data security, data protection, data recovery

## Abstract

**Background:**

After the Great East Japan Earthquake in 2011, backup systems for clinical information were launched in Japan. The system in Miyagi Prefecture called the Miyagi Medical and Welfare Information Network (MMWIN) is used as a health information exchange network to share clinical information among various medical facilities for patients who have opted in. Hospitals and clinics specializing in chronic renal failure require patients’ data and records during hemodialysis to facilitate communication in daily clinical activity and preparedness for disasters.

**Objective:**

This study aimed to facilitate the sharing of clinical data of patients undergoing hemodialysis among different hemodialysis facilities.

**Methods:**

We introduced a document-sharing system to make hemodialysis reports available on the MMWIN. We also recruited hospitals and clinics to share the hemodialysis reports of their patients and promoted the development of a network between emergency and dialysis clinics.

**Results:**

In addition to basic patient information as well as information on diagnosis, prescription, laboratory data, hospitalization, allergy, and image data from different facilities, specific information about hemodialysis is available, as well as a backup of indispensable information in preparation for disasters. As of June 1, 2021, 12 clinics and 10 hospitals of 68 dialysis facilities in Miyagi participated in the MMWIN. The number of patients who underwent hemodialysis in Miyagi increased by more than 40%.

**Conclusions:**

Our backup system successfully developed a network of hemodialysis facilities. We have accumulated data that are beneficial to prevent the fragmentation of patient information and would be helpful in transferring patients efficiently during unpredictable disasters.

## Introduction

Patients undergoing hemodialysis (HD) require collaborative team care, not only because their renal function is poor but also because they are vulnerable to various comorbidities [1,2]. Since an increase in the number of comorbidities makes care for patients more complex, multiple medical facilities may need to share patient information, and diverse specialist expertise may be required for patient management. However, sharing clinical information among different facilities is not an easy task because most dialysis clinics and hospitals do not have the same electronic health record (EHR) or do not have a health information exchange (HIE). Furthermore, the EHR and HD systems are rarely integrated. Such fragmentation of clinical information becomes a more serious concern during natural disasters such as earthquakes, tsunamis, and hurricanes. In 2011, the Great East Japan Earthquake (GEJE) hit Miyagi Prefecture [3,4] and damaged many facilities, including HD clinics and hospitals, resulting in the transfer of patients undergoing HD from Miyagi to other distant places [5]. At that time, the importance of patient information was recognized through experience. In addition, Katrina, a hurricane that hit the United States in 2005, caused similar situations in which many patients could not receive optimal dialysis treatments at alternate dialysis institutions because of a lack of medical records and information about their dialysis regimen [6]. Thus, sharing patient information is critical for disaster preparedness.

After the GEJE, backup systems of clinical data were developed in many places in Japan, and Miyagi Prefecture launched the Miyagi Medical and Welfare Information Network (MMWIN), which saves patient clinical information obtained from medical facilities, including hospitals, clinics, pharmacies, and nursing homes [7]. Moreover, the MMWIN is now used as an HIE to share clinical information, such as patients’ basic information, history of diagnosis, prescription data, laboratory test data, and hospitalization data, among more than 800 facilities in Miyagi Prefecture [8]. This study aimed to facilitate the sharing of clinical data of patients undergoing HD among HD facilities.

## Methods

### The MMWIN System and Data Storage

The system used in MMWIN was explained in detail in our previous paper [7]. Briefly, the main system of the MMWIN consists of backup storage, a portal server to manage patients’ ID registration information, the gateway that receives clinical data from facilities and stores them in the storage, and viewer applications. A standard storage format, Standardized Structured Medical Information eXchange version 2 (SS-MIX2), which is authorized by the Ministry of Health, Labour and Welfare of the Japanese government, enables the collection of clinical information from different vendor systems and is commonly used in several national projects to store and use large amounts of clinical data in Japan [9-12]. The storage format is divided into two categories. The first is the standardized storage, including standard clinical data in a standard form (Health Level 7 [HL7] v2.5), such as basic patient data, prescriptions, and laboratory data. The second is the extension storage that includes the remaining data not stored in the standard storage [12].

Computers in the MMWIN-affiliated facilities are connected to the MMWIN server via a virtual private network (MMWIN secure network). Clinical data from the hospital information system (HIS; or other information systems available in clinics, pharmacies, or nursing care homes) are transferred to SS-MIX2–formatted XML/HL7 files via gateways.

Opt-in for patients to register with the MMWIN includes a unique MMWIN ID number, which is connected to a different ID number in each facility, thereby permitting the sharing of patient information. Finally, these data are available through a browser-based SS-MIX2 viewer application, Human Bridge (Fujitsu). Thus, in each facility, the clinical information of all patients in the facility can be seen on MMWIN, which allows clinicians to check clinical data even when their own information system is out of order.

### Data Transfer of HD Reports

**Figure 1:**
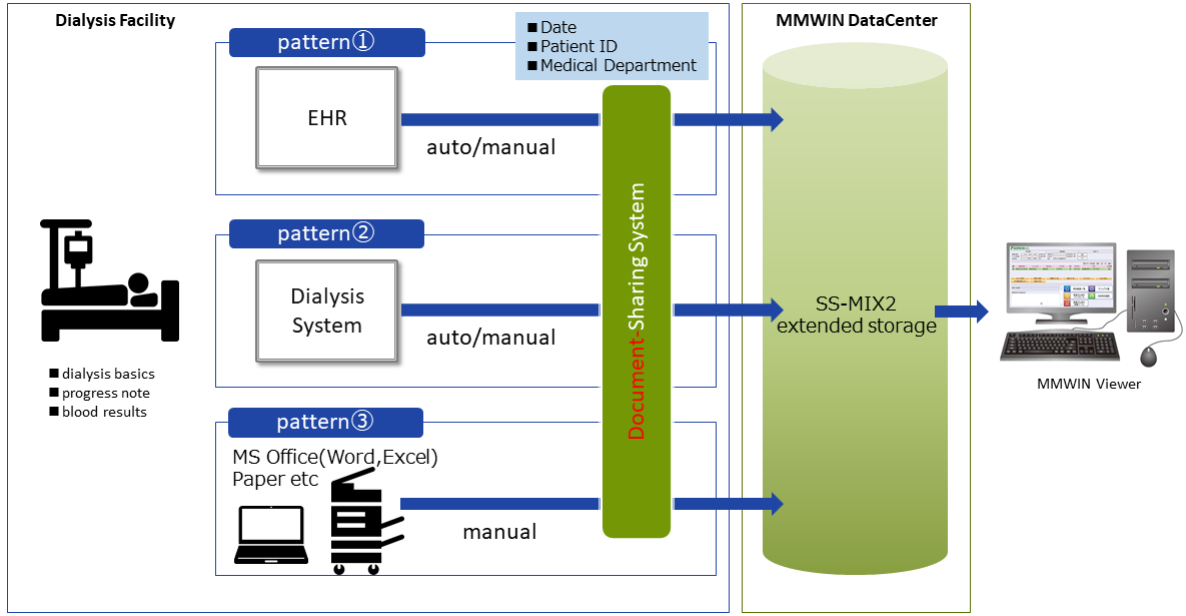
A scheme of connection between the MMWIN data center and the hemodialysis (HD) report in each facility. Three sources of HD information from facilities are shown: HD reports integrated with the hospital information system and transferred to the SS-MIX2 center in the MMWIN; HD reports released from the dialysis system and transferred to the SS-MIX2 center in the MMWIN; and a manual transfer of HD data via our document-sharing system. EHR: electronic health record; MMWIN: Miyagi Medical and Welfare Information Network; MS: Microsoft; SS-MIX2: Standardized Structured Medical Information eXchange version 2.

Since there is no standard format for HD reports, we started storing the reports in extension storage using Docuworks (FUJIFILM Business Innovation Corp.), which enables the transfer of HD reports from the facilities to the MMWIN center. Figure 1 shows the scheme of the system for sharing HD reports between the MMWIN and facilities.

There are three ways to transfer HD information from facilities. First, HD reports could be integrated into the HIS and automatically or manually transferred to the SS-MIX2 center in the MMWIN. Second, HD reports could be released from the dialysis system and automatically or manually transferred to the SS-MIX2 center in the MMWIN. Third, a facility that still uses a paper record of HD or the original format using Word or Excel (Microsoft Corporation) can transfer the data manually via our document-sharing system. Through Docuworks, these files with additional necessary patient information, such as patient ID and labels as HD reports, are converted to SS-MIX2 format and are transferred to the SS-MIX2 extension storage in MMWIN through the facility’s gateway and MMWIN secure network.

### Ethics Approval

The project was approved by the administrative boards of MMWIN on December 25, 2015.

### System Promotion

To promote the use of the system, we tried to encourage the development of a network within the clinical communities of HD clinics and hospitals. We asked nephrologists in Miyagi Prefecture to use this system and explained the benefits, such as being paperless and reducing the work burden of medical staff in HD facilities, through information brochures, personal meetings, and seminars since 2016. We also encouraged patients undergoing HD to provide consent to share their clinical information including prescription, laboratory data, and HD reports with the other HD facilities.

### Statistical Analysis

A chi-square test and the Welch *t* test were used for statistical analysis using R 3.6.0 (R Foundation for Statistical Computing). Two-sided *P* values <.05 were considered significant.

## Results

**Figure 2:**
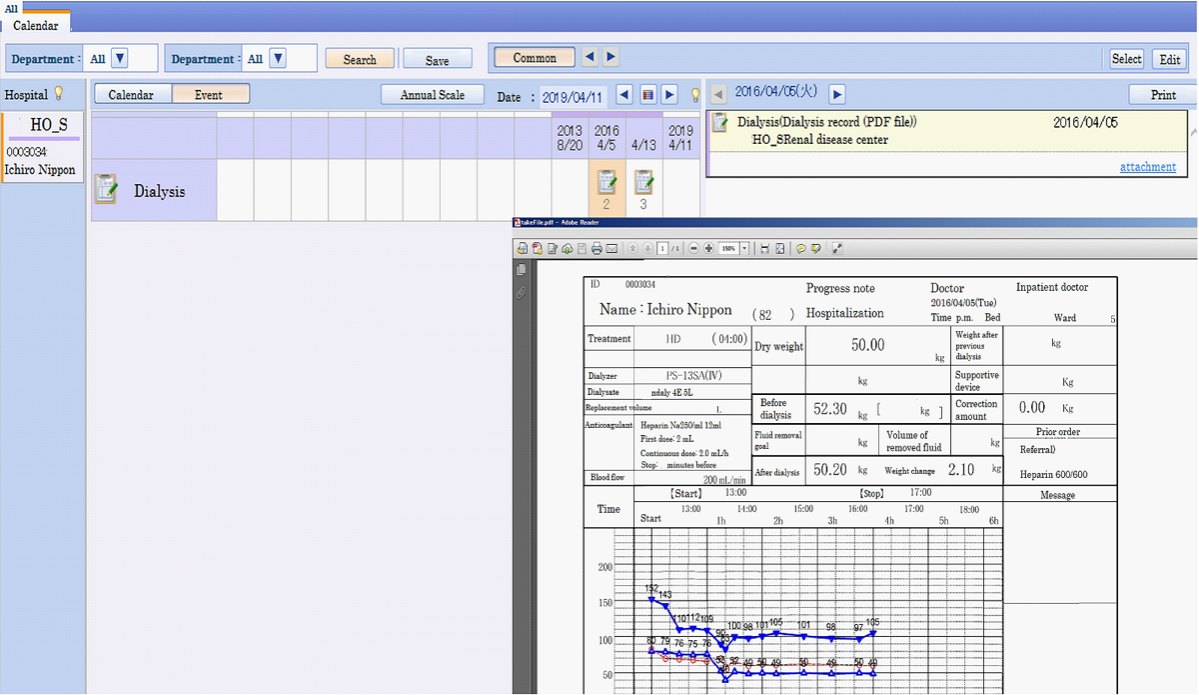
A screenshot of the Miyagi Medical and Welfare Information Network viewer and hemodialysis report. Users can access information on dialysis regimen, the latest body weight, and vital signs by this view.

We introduced a document-sharing system to make HD reports available on the MMWIN. Figure 2 shows a screenshot of the HD report on the MMWIN. MMWIN users can refer to data regarding HD as well as a variety of information, such as basic patient information, diagnosis, prescription, laboratory data, hospitalization, allergy information, and image data from different facilities. When a user clicks the icon, detailed information is obtained. Next, we compared the amount of information available on the MMWIN to the items that were required during a disaster, as recommended in previous reports [13,14]. Table 1 shows that 39 items are available in the MMWIN, and these covered 92% (23/25) of recommendation 1 [13] and 100% (19/19) of recommendation 2 [14].

**Figure 3:**
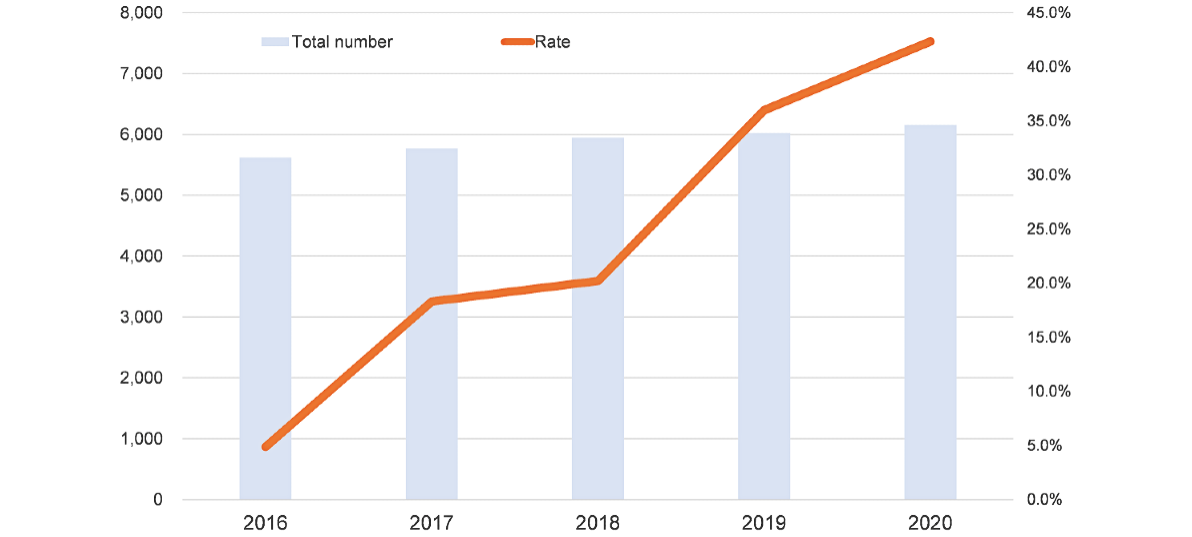
The rate of opt-in patients undergoing hemodialysis in the Miyagi Medical and Welfare Information Network and the number of patients undergoing hemodialysis in Miyagi Prefecture. The bars show the total number of patients undergoing hemodialysis in Miyagi. The line chart represents the ratio of opt-in patients to all patients undergoing hemodialysis in Miyagi.

According to the network linkage of patient transfer between core hospitals and HD facilities, we recruited hospitals and clinics sharing HD reports and promoted the development of a network between emergency hospitals and dialysis clinics. As of June 1, 2021, 12 clinics and 10 hospitals of the 68 dialysis facilities in Miyagi participated in the MMWIN. The basic characteristics of the facilities with or without HD are shown in Table 2. There were no significant differences among them, except for the number of beds in clinics, with a smaller number of beds in the facilities using the MMWIN-HD system (*P*=.02). We also asked patients for their consent to share clinical information among facilities. Figure 3 shows the rate of opt-in patients (line chart) and the total number of all patients undergoing HD in Miyagi Prefecture (bar chart). The number of opt-in patients increased to more than 40% of all patients who underwent HD in Miyagi Prefecture after we began recruitment.

**Table 1:** Available information about dialysis in the MMWIN and recommended data items for disaster preparation.

	Recommendation 1 [13]	Recommendation 2 [14]	MMWIN^a^
Institution		✓^b^	✓
Cause of renal failure		✓	✓
Diabetic yes/no		✓	✓
Dialyzer	✓	✓	✓
Anticoagulant		✓	✓
Hours of dialysis	✓	✓	✓
Times per week	✓		✓
Date of previous HD^c^	✓	✓	✓
Primary diagnosis	✓		✓
Allergies	✓	✓	✓
Medications	✓	✓	✓
Past medical history	✓	✓	✓
Center HD	✓		✓
Home HD	✓		
CAPD^d^	✓		✓
CCPD^e^	✓		✓
HDF^f^ no/pre/post		✓	✓
Dialysis prescription	✓	✓	✓
Usual dialysis machine		✓	✓
Vascular access	✓	✓	✓
Dialysate	✓		✓
Start time			✓
End time			✓
BP^g^ before HD			✓
BP after HD			✓
Weight before HD			✓
Weight after HD			✓
Dry weight	✓	✓	✓
Body temperature			✓
Average weight gain	✓	✓	✓
Comments	✓	✓	✓
Heparinization	✓		✓
Needle size	✓	✓	✓
Blood flow rate	✓		✓
Reuse^h^	✓		
Lidocaine	✓		✓
HBsAg^i^	✓		✓
Blood type	✓		✓
Laboratory data		✓	✓

^a^MMWIN: Miyagi Medical and Welfare Information Network.

^b^Indicates this item is present in the recommendations or MMWIN.

^c^HD: hemodialysis.

^d^CAPD: continuous ambulatory peritoneal dialysis.

^e^CCPD: continuous cycling peritoneal dialysis.

^f^HDF: hemodiafiltration.

^g^BP: blood pressure.

^h^Reuse is not allowed in Japan

^i^HBsAg: hepatitis B surface antigen.

**Table 2:** The characteristics of hemodialysis facilities participating in the Miyagi Medical and Welfare Information Network.

Facility		Attending (n=22), n			Not attending (n=46), n	
		Clinic	Hospital	Clinic		Hospital
**Fund**						
	Public	0	5		0	9
	Private	12	5		26	11
**Area**						
	Coast	1	2		3	3
	North	3	1		6	7
	South	4	1		3	2
	Central	4	6		14	8
Beds, mean (SD)		1.6 (5.3)	279.3 (320.4)	7.7 (9.1)		306.8 (182.3)
Beds for HD^a^, mean (SD)		51.8 (22.1)	40.1 (19.2)	37.4 (13.0)		28.8 (17.3)
Doctors, mean (SD)		2.1 (1.1)	155.1 (396.1)	2.0 (1.1)		67.8 (68.7)
HD patients, mean (SD)		118.0 (66.5)	95.9 (55.7)	87.9 (51.1)		70.8 (55.5)

^a^HD: hemodialysis.

## Discussion

### Principal Results

We developed a system for sharing HD reports and other clinical information among medical facilities and enhancing their connection networks. This would be helpful when the condition of patients undergoing HD is aggravated and when they need to be transferred to other hospitals. It would also be effective for the improvement of medical staff members’ work burden in the view of a paperless environment where they do not have to send HD reports by fax or mail to other facilities. Another merit is that patient information can be made available during a disaster, resulting in prompt extraction of patient information even in a chaotic situation. Lastly, sharing patient information would be very helpful for collaborative care, especially among general care practitioners and specialists, including nephrologists. An HIE can be a good tool to support clinical decision-making and ensure continuity of care, especially for complex medical problems.

In catastrophic situations, like the GEJE in 2011, the clinical information on MMWIN can be accessed from different facilities to acquire patient clinical data when needed. Since the MMWIN system is independent of the HIS in each facility, the information can be accessed even if the HIS is unavailable. Medical staff members can refer to medication and laboratory results and input clinical notes on the MMWIN system. Therefore, even when an HIS is down, medical staff can conduct HD as usual according to patient records on MMWIN.

### Comparison With Prior Work

Access to prior clinical data from different facilities is important for continuing appropriate treatment in the community. A fully interoperable EHR or HIE would also be helpful in maintaining the flow of critical patient information. In particular, for the care of patients undergoing HD, the information would reduce the risks of HD and prevent aggravation of renal function. In our system, we introduced a document management system for several measures to transfer HD reports from an EHR, dialysis systems, or original documents. Although specific information systems were launched elsewhere [15,16], we successfully integrated the sharing of HD reports into the main HIE system without the high cost of additional implementation. This system is based on document sharing. As the next step, digital data transfer is desirable for using clinical data, such as copying and pasting to avoid tedious record keeping by medical staff in daily clinical activity or for research purposes when researchers obtain informed consent from patients for secondary use. In addition to HD, since our document-sharing system is used in various situations, such as reference letters when a patient is transferred, notes to doctors from pharmacists when patients have questions regarding medication at the pharmacy, and sharing documents between care workers and doctors, this system can be used in different fields as a digital transformation tool.

The future of interoperability may lie in using novel standards to transfer clinical data more easily between an HIS and mobile devices, such as personal health records (PHRs). One such standard may be based on the Fast Healthcare Interoperability Resource (FHIR) [17]. We have already attempted to transmit clinical data using the FHIR for PHRs [18]. It could also be applied to HD reports soon. In addition, this system is used for care coordination between primary care physicians (PCPs) and nephrologists to manage the associated complex chronic conditions of patients undergoing HD. This could improve the management of patients with end-stage renal disease [19]. Our previous study revealed that an HIE is a good tool to improve patient prognosis, with collaboration between PCPs and specialists [8].

One of our major concerns in patient management is preparedness for disasters. During a disaster, HD facilities sometimes cannot supply the usual HD environment, resulting in HD at alternate dialysis locations without patients’ medical records or information about their dialysis regimen being available. Therefore, we checked whether the available information on the MMWIN could cover the necessary data items during a disaster for dialysis-specific disaster preparedness [13,14,20]. Thus, as shown in Table 1, we covered almost all the recommendations. Available data provided in MMWIN include the latest body weight and vital sign on regular HD, which are essential information for optimal and secure dialysis management. Although it is recommended that the patients themselves always have basic information of their own for disaster preparedness, this is not easy because older people comprise two-thirds of patients undergoing dialysis in Japan [21].

### Limitations

This study had some limitations. First, we did not include all HD facilities in Miyagi. Although we managed to enroll 22 HD facilities joining the MMWIN and more than 40% of patients undergoing HD in Miyagi were registered, HD records from dialysis facilities cannot be shared with other dialysis facilities that do not have access to the MMWIN. It would be difficult for small facilities to implement the system without the support of public budgets. Encouragement by the local government through stronger incentives to both facilities and EHR vendors is needed to implement the MMWIN system. There were no significant differences among facilities, except for the number of beds in clinics, with fewer beds in the facilities within the MMWIN system (*P*=.02; Table 2). In the survey, most clinics with fewer beds were HD-specific facilities that did not have admission beds for inpatients, only for outpatients with HD. Table 2 shows that the HD-specific facilities favored MMWIN systems over other facilities, mainly because of our document-sharing system for patients undergoing HD. This indicates that the HD-specific clinics for only HD outpatients favored using the MMWIN-HD system to share patient information with hospitals, which would accept patients when their condition worsened. In addition to HD reports, the document systems are available for sharing reference letters or notes among hospitals, between hospitals and clinics, between doctors and pharmacists, and between hospitals and nursing care homes. As well as HD facilities, we believe that all health care facilities should make an effort to share patient information across all care settings. Another potential benefit for such a network may be its use to guide medical decisions based on individual patient characteristics (personalized medicine) rather than averages over a whole population. Personalized medicine in patients with kidney disease is lagging behind the other medical disciplines as most randomized controlled trials are currently excluding patients with kidney disease. Second, we have not encountered a disaster that requires HD records to be shared among facilities. This is fortunate, but there is a requirement to test the system with more practical simulations for possible disasters.

Additionally, we did not have a wide range of training exercises among different facilities to simulate large disasters. Support from patients and many facilities throughout the prefecture would be practical. A plan for a wide range of training exercises is needed in the near future. However, the staff members in facilities attending MMWIN have already implemented daily use of this system in clinical activities. This habit can be helpful, even during disaster situations.

### Conclusions

In conclusion, our backup system successfully developed a network of HD facilities. We have accumulated data that are beneficial to prevent the fragmentation of patient information and would be useful to transfer patients efficiently as preparedness for unpredictable disasters.

## Abbreviations

EHR: electronic health record
FHIR: Fast Healthcare Interoperability Resource
GEJE: Great East Japan Earthquake
HD: hemodialysis
HIE: health information exchange
HIS: hospital information system
HL7: Health Level 7
MMWIN: Miyagi Medical and Welfare Information Network
PCP: primary care physician
PHR: personal health record
SS-MIX2: Standardized Structured Medical Information eXchange version 2
